# Importance of pH Homeostasis in Metabolic Health and Diseases: Crucial Role of Membrane Proton Transport

**DOI:** 10.1155/2014/598986

**Published:** 2014-09-11

**Authors:** Wataru Aoi, Yoshinori Marunaka

**Affiliations:** ^1^Laboratory of Health Science, Graduate School of Life and Environmental Sciences, Kyoto Prefectural University, Kyoto 606-8522, Japan; ^2^Departments of Molecular Cell Physiology and Bio-Ionomics, Graduate School of Medical Science, Kyoto Prefectural University of Medicine, Kyoto 602-8566, Japan; ^3^Japan Institute for Food Education and Health, St. Agnes' University, Kyoto 602-8013, Japan

## Abstract

Protons dissociated from organic acids in cells are partly buffered. If not, they are transported to the extracellular fluid through the plasma membrane and buffered in circulation or excreted in urine and expiration gas. Several transporters including monocarboxylate transporters and Na^+^/H^+^ exchanger play an important role in uptake and output of protons across plasma membranes in cells of metabolic tissues including skeletal muscle and the liver. They also contribute to maintenance of the physiological pH of body fluid. Therefore, impairment of these transporters causes dysfunction of cells, diseases, and a decrease in physical performance associated with abnormal pH. Additionally, it is known that fluid pH in the interstitial space of metabolic tissues is easily changed due to little pH buffering capacitance in interstitial fluids and a reduction in the interstitial fluid pH may mediate the onset of insulin resistance unlike blood containing pH buffers such as Hb (hemoglobin) and albumin. In contrast, habitual exercise and dietary intervention regulate expression/activity of transporters and maintain body fluid pH, which could partly explain the positive effect of healthy lifestyle on disease prognosis.

## 1. Introduction

Body fluid pH is determined by the content of protons (H^+^) generated from organic acids produced in living cells. Lactic acid (lactate^−^/H^+^) is a typical proton source and is involved in the regulation of physiological pH. In metabolic tissues such as skeletal muscle and adipose tissue, the glycolytic anaerobic metabolism mediates the conversion of glucose and glycogen into lactic acid. Because the pKa of lactic acid is 3.80, it is immediately dissociated into lactate (lactate^−^) and protons under physiological conditions, resulting in reduced intracellular pH. Pyruvic acid (pyruvate^−^/H^+^), an intermediate metabolite in the glycolytic system, is also a source of protons, although it generates much less protons compared to lactic acid. In addition, metabolites such as ketone bodies also act as proton sources. Beta-hydroxybutyric acid (beta-hydroxybutyrate^−^/H^+^), a typical ketone body, is generated as a result of fatty acid metabolism in the liver and is also dissociated into beta-hydroxybutyrate anions and protons, leading to the reduction of intracellular pH.

**Figure 1:**
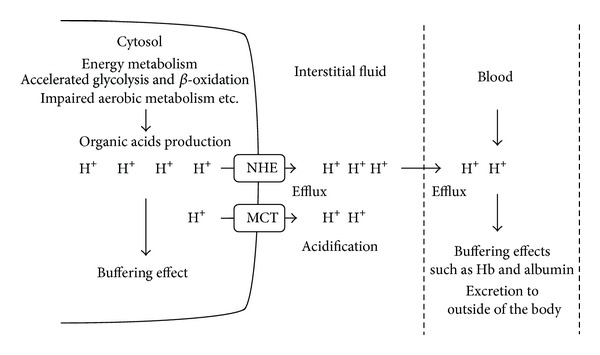
Proton production and its transporting kinetics in intracellular and extracellular fluid in metabolic tissues. The production of organic acids such as lactic acid and ketone bodies is accelerated by elevating glycolytic anaerobic metabolism and *β*-oxidation in metabolic cells. Body fluid pH is strictly maintained by buffering systems, efflux across plasma membrane, and acid excretion. Monocarboxylate transporter (MCT) and Na^+^/H^+^ exchanger (NHE) contribute to proton extrusion from the cytosol to the extracellular space. In contrast to intracellular fluid and blood containing pH buffers such as Hb (hemoglobin) and albumin, the interstitial fluid pH could be easily reduced by acid stress owing to the limited availability of the buffering factors such as proteins.

The intracellular pH in most living cells is alkaline compared to the pH generated by protons that are transported passively through the plasma membrane by electrochemical forces. In addition to buffering systems such as the bicarbonate-carbonate system, protein-proton binding, and phosphoric acid, several membrane transporters are responsible for proton removal from the cytosol and play important roles in maintaining the alkaline pH in cells (Figure 1). In most mammalian cells, H^+^-monocarboxylate cotransporters (MCTs) participate in the transport of monocarboxylic acids such as lactate, pyruvate, beta-hydroxybutyrate, and acetoacetate across the cellular membrane by cotransporting protons and monocarboxylate anions [1–3]. Other transporters such as the Na^+^/H^+^ exchanger (NHE) and bicarbonate-dependent exchanger also contribute to proton extrusion from the cytosol to the extracellular space [4, 5]. This review focuses on the critical role of the membrane transport system of protons in regulation of intracellular and extracellular fluid pH and its importance in maintaining physiological homeostasis and preventing diseases development.

## 2. Proton Transport across the Plasma Membrane in pH Regulation

Regulation of body fluid pH is one of the most important physiological functions of homeostasis, because activity of most chemical reactions via enzyme proteins is dependent on fluid pH. To maintain homeostasis of body fluid pH, various buffering systems are utilized in addition to proton excretion from the cytosol to the extracellular space and ultimately outside of the body. However, if production of organic acid is elevated or the buffering and excretion systems are impaired, body fluid turns acidic, leading to abnormal conditions. A typical example is elevation of lactic acid production in skeletal muscle in response to strenuous exercise, which leads to body fluid acidosis, preventing muscle contraction [6, 7]. Proton transport across the plasma membrane of muscle cells is important for maintaining the appropriate intracellular pH. Skeletal muscle is a major metabolic organ that generates acids, in particular during contraction. Strenuous muscle contractions can cause a drastic reduction in intramuscular pH to −6.5 with accumulation of more than 40 mM lactate [6–8], regardless of cellular buffering capacity. Several studies have shown that intracellular pH is reduced during muscle contraction and has a delayed recovery to basal conditions during the recovery phase in the absence of proton transporters [9]. This delay suggests that proton transporters play a key role in maintaining pH homeostasis. Indeed, the function of proton transporters is involved in the capacity for pH maintenance [9, 10]. In particular, over 80% of intracellular proton is transported through lactate cotransport in contracting muscle, although remaining parts are transported through NHE and bicarbonate-depending transport [8, 11]. The liver, another organ that is closely associated with the metabolism of organic acids, generates ketone bodies (i.e., acetoacetic and *β*-hydroxybutyric acids), metabolizes lipids, and converts lactate to glucose via gluconeogenesis. Therefore, this organ generates acidic conditions [12–14] and intracellular pH should be maintained by proton extrusion along with buffering function.

MCTs, a part of the solute carrier (SLC) 16 that contains 14 members in total, play a crucial role in proton transport across the plasma membrane by cotransporting monocarboxylates. Each isoform has different transport kinetics and is specifically located on a particular subcellular site. It has been shown that MCT1–MCT4 transport aliphatic monocarboxylates such as lactate, pyruvate, and ketone bodies [2] and that the direction of transport across the plasma membrane in a 1 : 1 manner is determined by the concentration gradients of protons and monocarboxylate both inside and outside of the cell [15–17]. Thus, these isoforms play important roles in proton transport maintaining intracellular pH. In particular, the expression of two MCT isoforms (MCT1 and MCT4) is associated with lactate disposal in muscles. MCT1 is highly expressed and located in both the sarcolemmal and the mitochondrial membranes of oxidative muscles [18–20]; on the other hand, MCT4 is predominantly located on the plasma membrane of glycolytic muscle and is assumed to contribute to lactate efflux [19, 21]. In contrast, MCT2 is mainly located on the membranes of liver cells and contributes to the extrusion of ketone bodies [22]. Other members of the family have different substrate specificities. For example, MCT6 has been shown to transport bumetanide, a diuretic drug [23], MCT 8 acts as a thyroid hormone transporter [24], MCT9 is a potential carnitine extrusion transporter [25], and MCT10 is identified as a low-affinity transporter of aromatic amino acids along with iodothyronines [26]. In addition, NHE is known as another major proton transporter that plays an important role in intracellular pH homeostasis by exchanging intracellular proton with extracellular Na^+^ using the chemical gradient between intra- and extracellular Na^+^ concentrations [4, 27]. Currently, 10 isoforms are known to exist in mammals. NHE1–NHE5 are located on the plasma membrane of their specific tissues, while NHE6–NHE9 are located on the membrane of subcellular organelles [27–29]. In particular, NHE1 has been recognized as a ubiquitous isoform and plays an important role in maintaining homeostasis in metabolic organs.

Proton transport across the plasma membrane is important for maintenance of intracellular and extracellular fluid pH. In particular, proton excretion and bicarbonate reabsorption are recognized as important function of renal tubules. Proton excretion into urine is mainly mediated by both proton-ATPase and NHE3 located on the apical plasma membrane of the proximal convoluted tubule participating in approximately 80% of bicarbonate reabsorption occurring in the whole kidney, acting as the major buffering system in blood [30, 31], which has also pH buffers such as Hb (hemoglobin) and albumin. Bicarbonate reacts with protons via catalytic carbonic anhydrase on the apical membrane and generates CO_2_. Then, it is transported into the blood by sodium-bicarbonate cotransporters on the basolateral side [32].

## 3. pH Disturbance and Disease Development

The normal physiological pH of mammalian arterial blood is strictly maintained at 7.40; blood has pH buffers such as Hb (hemoglobin) and albumin. A decrease of more than 0.05 units from the normal pH results in acidosis. The body fluids of diabetic patients are chronically acidic and exhibit characteristic ketoacidosis caused by an increased level of ketone bodies in the blood [33, 34]. Insulin resistance in metabolic tissues such as skeletal muscle, adipose tissue, and the liver accelerates the utilization of lipids as an energy substrate instead of glucose. Excess lipolysis caused by impaired glucose metabolism leads to free fatty acids in circulation, which facilitate hepatic gluconeogenesis by the oxidation of fatty acids resulting in large quantities of ketone bodies. This further accelerates proton overloads, leading to the metabolic ketoacidosis found in diabetic patients. Such acidic conditions prevent the activity of metabolic enzymes such as phosphofructokinase and further accelerate the progression of pathological conditions [33–35]. Acidic conditions can also result in physical fatigue of diabetic patients. Therefore, maintaining normal pH is important for physiological homeostasis.

It has been suggested that loss of function of MCTs causes a change of body fluid pH. Several point mutations of the MCT gene have been shown to affect both specificity and transport activity. The spontaneously occurring mutation of arginine 306 to threonine in domain 8 of MCT1 resulted in reduced transport activity [36]. In addition, it has been shown that subjects who have mutations in MCT1 cDNA have drastically lower transport rates and a delayed decline of blood lactate after exercise [37, 38]. Healthy subjects feel severe chest pain and muscle cramping after strenuous exercise, along with a defect in lactate efflux from muscle. Furthermore, many amino acid differences that are not attributable to polymorphisms are found in MCT1 obtained from muscle tissues in these subjects [37, 39]; thus, mutations in MCT1 are related to physical fatigue and exercise performance. MCT dysfunction may lead to metabolic disorder. Indeed, lower level expression of MCT1 and MCT4 is found in the skeletal muscle of obese rats compared to normal rats [40]. In addition, the activity of lactate transport in muscle is also decreased by both denervation and aging [41, 42]. A significant negative correlation between the level of circulating lactate and degree of insulin sensitivity is found in humans [43], suggesting that lower lactic acid disposal caused by reduction of MCT function is associated with insulin resistance.

## 4. Interstitial Fluid pH and Disease Development

Body fluid acidosis could also contribute to the development of metabolic diseases. Our recent study indicates that before the development of diabetic symptoms the interstitial fluid pH in ascites and metabolic tissues of Otsuka Long-Evans Tokushima Fatty (OLETF) rats is lower than the normal pH (7.40) [44]. The buffering capacity is relatively high in the cytosol and blood but low in the interstitial fluid due to limited buffering factors such as proteins [45, 46]. Therefore, interstitial fluid pH in metabolic tissues easily changes (Figure 1) and may contribute to the onset of insulin resistance. We have shown the inhibitory effect of extracellular pH on the insulin signaling pathway in the L6 rat myotube. The phosphorylation level and binding affinity to insulin of insulin receptors were significantly diminished in media with low pH [47]. In addition, the levels of Akt phosphorylation, a downstream of the insulin receptor, are also decreased in low pH media, along with a reduction in glucose uptake. These* in vitro* observations support the hypothesis that lower extracellular pH may cause insulin resistance in skeletal muscle cells. Other studies [48–50] have suggested a close correlation between organic acid production and insulin sensitivity in both type 2 diabetes patients and healthy subjects. In a cross-sectional study of over 1,000 subjects [48], it has been demonstrated that body weight and waist circumference have a negative correlation with both insulin sensitivity and urine pH. Patients with metabolic syndrome have also reported a significantly lower pH of 24 h urine compared to the normal subjects and a negative correlation between the mean 24 h urine pH and the number of metabolic syndrome abnormalities [49, 50]. It has been suggested that lower levels of serum bicarbonate and higher levels of anion gap resulting from metabolic acidosis are associated with lower insulin sensitivity [51]. Hyperlactacidemia is found in patients with obesity and type 2 diabetes [43], which supports the strong relationship between acidic condition and insulin sensitivity. Even in healthy subjects, acids level could be an independent risk factor for the development of type 2 diabetes [52].

Insulin resistance is one of the major symptoms of metabolic disorders and is frequently associated with hypertension, high blood glucose levels, visceral obesity, and dyslipidemia. Insulin resistance also causes type 2 diabetes and plays a key role in developing cancer and cardiovascular disease. Thus, pH abnormalities can cause abnormal metabolic regulation in a predisease state. We recently found an observation that the interstitial pH around the hippocampus, an important region for memory [53], is lower in diabetic OLETF rats (26 weeks of age) than in normal Wistar rats [54]. It has been reported that diabetic patients have a high risk of developing dementia and Alzheimer's disease [55] and may experience defective memory functions. The insulin action is required for neuronal survival within the central nervous system [56]. Fluctuating glucose levels resulting from defective insulin have been suggested to lead to apoptosis, energy starvation, formation of neuritic plaques and neurofibrillary tangles, hallmark lesions of Alzheimer's disease, and altered acetylcholine levels in the hippocampus [57, 58]. Therefore, we indicate that maintenance of the interstitial fluid pH within the normal range or the recovery of the interstitial pH to the normal range could be one of the most important factors in developing molecular and cellular therapies for metabolic brain disorders.

## 5. pH Regulation by Diet and Exercise Intervention

The maintenance of pH in metabolic organs is achieved through various regulatory systems. Physical exercise and appropriate diet contribute to pH homeostasis. Habitual exercise adaptively accelerates the entry of fatty acids both from the plasma into the muscle cell and from the cytosol into the mitochondria, while also enhancing Krebs cycle function in the resting state. Their actions are caused by elevation of activity and expression of related enzymes in skeletal muscles [59–61]. Since the energy consumed in muscle during exercise is mainly supplied by carbohydrates and lipids, the exercise-induced lipid utilization may decrease the energy obtained from carbohydrates, further decreasing the lactate/proton production, or lactic acidosis. In addition, circulating and intramuscular buffering capacities are improved via habitual exercise increasing proteins, amino acids, and phosphate [62–64]. Peripheral circulation is also improved through vasodilation caused as a physiological adaptation to exercise [65], which further facilitates the proton washout. In particular there is evidence suggesting that excretion of protons from the cytosol to the extracellular space or into circulation via transporters located on the plasma membrane contributes to the prevention of intracellular acidosis. It has been reported that exercise training increases the MCT1 and MCT4 levels in the skeletal and cardiac muscle of humans and animals [66–68]. Although the regulation of MCT expression levels is not clearly understood, it has been suggested that protein kinases A and B are involved in the regulation of MCT expression [69] as an adaptation mechanism, which may be mediated by an increase in lactate movement across the membrane. In addition, our recent study has reported that MCT1 content in erythrocyte membranes is elevated by exercise training in rats [70, 71]. A proportion of the lactate released from skeletal muscles into the plasma is taken up by erythrocytes. The mature erythrocytes generate ATP only through the glycolytic pathway, since they have no mitochondrial machinery. Thus, erythrocytes cannot utilize lactate produced as a respiratory fuel and this necessitates the release of lactate into the plasma via MCT1 [72]. However, one of the most important roles of erythrocytes is to distribute released monocarboxylates by taking up monocarboxylates, since erythrocytes produce much less lactate than other tissues. Based on the results of our* in vitro* study, the skeletal muscle may be entirely dependent on MCT1-mediated lactate uptake by erythrocytes to maintain pH homeostasis [71]. In addition, there is a high correlation between the athletic performance of horses and their erythrocyte lactate concentrations after racing [73]. Therefore, efficient proton transport via MCTs induced by habitual exercise may contribute to the improvement of insulin sensitivity and muscle fatigue caused by lowered pH.

It is well known that adequate diet is important for controlling pathological conditions in patients with metabolic disorders. In addition, intervention studies in humans have reported that several bioactive factors included in foods such as antioxidants [74–77] and n-3 unsaturated fatty acids [78, 79] improve energy metabolism. Additional factors such as carotenoids, alpha lipoic acids, amino acids/peptides, and minerals may also offer preventive or therapeutic effects to combat hyperglycemia and several animal and culture studies have demonstrated their efficacy in improving insulin sensitivity [80–84]. The effects of these nutrients are only beneficial when administered in combination. In contrast to the successful application of dietary approaches or combined nutrients [85–87], various types of intervention studies using single nutrients have failed to clarify their beneficial action on cardiovascular risk and insulin resistance [88, 89]. Therefore, administration of multiple nutrients is considered more effective when compared to administration of a single bioactive factor. Propolis, a natural product derived from the plant resins collected by honeybees, contains various types of compounds including polyphenols, phenolic aldehydes, sesquiterpene quinones, coumarins, amino acids, steroids, and inorganic compounds [90] and has been reported to reduce the metabolic defects caused by abnormal blood glucose and insulin in young (18 weeks of age) OLETF rats [42] characterized by hyperphagia, obesity, decreased glucose infusion rate in a euglycemic clamp at 16–18 weeks of age, hyperinsulinemia around 25 weeks of age responding to an intravenous glucose infusion, and developing type 2 diabetes [91, 92]. Thus, our study indicates that propolis has a beneficial and preventive action on type 2 diabetes mellitus at early stages developing insulin resistance. Further, we have obtained evidence that intake of propolis elevates the pH of ascites and metabolic tissues compared with normal diet, indicating that dietary propolis diminishes production of organic acids or increases buffering capacity in those tissues. Therefore, propolis may be a useful compound to improve insulin sensitivity via prevention of metabolic acidosis. The molecular mechanism of how propolis improves interstitial pH is unclear, and we should strive to better understand the mechanism of this bioactive supplement.

## 6. Conclusion

Membrane transport of protons is required for preventing acidic states of body fluid, maintaining physical performance, and improving metabolic impairments. In contrast to the intracellular and blood pH, interstitial fluid pH can easily be reduced by acid stress. This can disturb homeostasis of the intracellular metabolism, leading to the development of metabolic diseases. However, detailed mechanisms including the involvement of membrane transport of protons responsible for the reduction of interstitial fluid pH are unknown. In addition, activity and expression of proton transporters such as MCT and NHE are easily altered by various changes in the cell environment. More studies are required to examine the detailed regulatory mechanisms of proton transporters, including gene expression, protein modification, and membrane trafficking, in addition to their contributions to metabolic homeostasis.
